# Natural history of Valentin’s rock lizard (Darevskia valentini) in Armenia

**DOI:** 10.24272/j.issn.2095-8137.2019.036

**Published:** 2019-07-18

**Authors:** Eduard Galoyan, Alisa Bolshakova, Manush Abrahamyan, Ruzanna Petrosyan, Valeria Komarova, Spangenberg Viсtor, Arakelyan Marine

**Affiliations:** 1 Zoological Museum of the Lomonosov Moscow State University, Moscow 125009, Russia; 2 Department of Zoology, Biological Faculty of Moscow State University, Moscow 119992, Russia; 3 Department of Zoology, Biological Faculty of Yerevan State University, Yerevan 0025, Armenia; 4 Severtsov Institute of Ecology and Evolution of the Russian Academy of Sciences, Moscow 119071, Russia; 5 Vavilov Institute of General Genetics, Russian Academy of Sciences, Moscow 119991, Russia

**Keywords:** *Darevskia valentini*, Reproduction, Population density, Skeletochronology, Home range, Seasonal activity

## Abstract

Valentin’s rock lizard (*Darevskia valentini*) is suggested to be the parent for several parthenogenetic species (e.g., *D. armeniaca*, *D. bendimahiensis, D. sapphirina*, and *D. unisexualis*) that evolved through hybridization. Complex evolutionary processes (including reticulate evolution) are occurring within the areas where Valentin’s rock lizard coexists with these and other rock lizards. Hence, a detailed biological specification of this species is important for understanding how vertebrates evolve. Valentin’s rock lizard is a long-lived (up to 9 years), small diurnal lizard with larger females than males, which is unlike other species of the genus. Their relatively large eggs and early reproduction period, which occurs just after emergence from winter shelters, are adaptations for living in a high elevation climate (higher than 2 000 m a.s.l.). Their body temperatures (31–32 °С) are comparable to body temperatures of rock lizards living in milder climates, though female body temperature is more dependent on substrate temperature and basking due to their lower activity than that found in males. Population density fluctuates from several individuals to several hundred per hectare and is not affected by parthenogen coexistence, although hybrids do occur in sexually biased populations where males are more common than females. The male home range is larger than that of females, though these home ranges broadly overlap. Prey is not limited in the mountain meadows and Valentin’s rock lizards feed on a great variety of arthropods. Infanticide occurs in high-density populations.

## INTRODUCTION

Rock lizards belong to the genus *Darevskia* (Arribas, 1999; Arribas et al., 2017) or the recently suggested genus *Caucasilacerta* (Busack et al., 2016), which comprises about 30 relatively small species, seven of which reproduce parthenogenetically. The lizards of this genus are distributed in the Caucasus Mountains, Armenian highlands, northern Iran, western Turkmenistan, eastern Turkey, and Crimean Peninsula. Due to hybrid speciation in this genus, Caucasian rock lizards are important model organisms for the study of evolutionary mechanisms (Danielyan et al., 2008; Darevsky, 1967; Freitas et al., 2016; Ryskov et al., 2017; Spangenberg et al., 2017; Tarkhnishvili et al., 2013). However, information on the ecological traits and requirements of most *Darevskia* species is limited. Their biological features were first partially described by Darevsky (1967), with other researchers since undertaking studies on niche differentiation (Tarkhnishvili et al., 2010), age estimation (Arakelyan, 2002; Kurnaz et al., 2017; Sergeev, 1937; Tsellarius & Tsellarius, 2009), foraging and prey composition (Darevsky & Danielyan, 1967; Lukina, 1963), and spacing and social behavior (Galoyan, 2010, 2013a; Tsellarius & Tsellarius, 2001; Tsellarius et al., 2016, 2017). However, there is still a general lack of information on the natural history of rock lizards. Moreover, our basic understanding of the underlying mechanisms of modern geographical distribution and evolutionary scenarios needs to be improved, in particular the specific ecological traits within the genus *Darevskia*.

Valentin’s rock lizard (*Darevskia valentini* (Boettger, 1892)) is a rock-dwelling lizard that mainly inhabits meadows at elevations between 1 900 and 3 110 m a.s.l. in the Small Caucasus, particularly in northern Armenia, southern Georgia, northern Iran, and eastern Turkey (Arakelyan et al., 2011;http://reptile-database.reptarium.cz, 2017). This taxon was previously described as *Lacerta saxicola terentjevi* (Darevsky, 1957) and then synonymized with the earlier suggested name *Lacerta saxicola valentini* (Darevsky, 1965). The high elevation and extreme climate have contributed to the distinct natural history of this species. Furthermore, this species is of interest due to its evolutionary role in the origin of several parthenogens, namely *D. armeniaca*, *D. bendimahiensis*, *D. sapphirina*,and *D. unisexualis* (Fu et al., 2000; Tarkhnishvili et al., 2016). Among mtDNA clades, according to their phylogeny (Ahmadzadeh et al., 2013; Murphy et al., 2000), this species belongs to the “rudis” clade. The hybrid origin of parthenogenetic rock lizards is not controversial (Darevsky et al., 1985; Murphy et al., 2000; Tarkhnishvili et al., 2017). It is suggested that *D. valentini* is the paternal species for those listed above. Triploid and even tetraploid hybrids between parental and parthenogenetic species have also been described previously for sympatric populations (Danielyan et al., 2008; Darevsky & Kupriyanova, 1985); however, their evolutionary role is still unrecognized. Hence, understanding the hybridization conditions, ecological preferences, and biological specification of the parental species is crucial for our understanding of the appearance of parthenogens.

The aims of this study were to consolidate data on the autecology of Valentin’s rock lizard from allopatric and sympatric populations and to describe the main biological traits of the species, which are important for predicting evolutionary output within rock lizards. Here we present considerable data compiled from different areas and years by our research team and provide a general overview of the ecological characteristics of Valentin’s rock lizard.

## MATERIALS AND METHODS

### Study object

**Figure 1:**
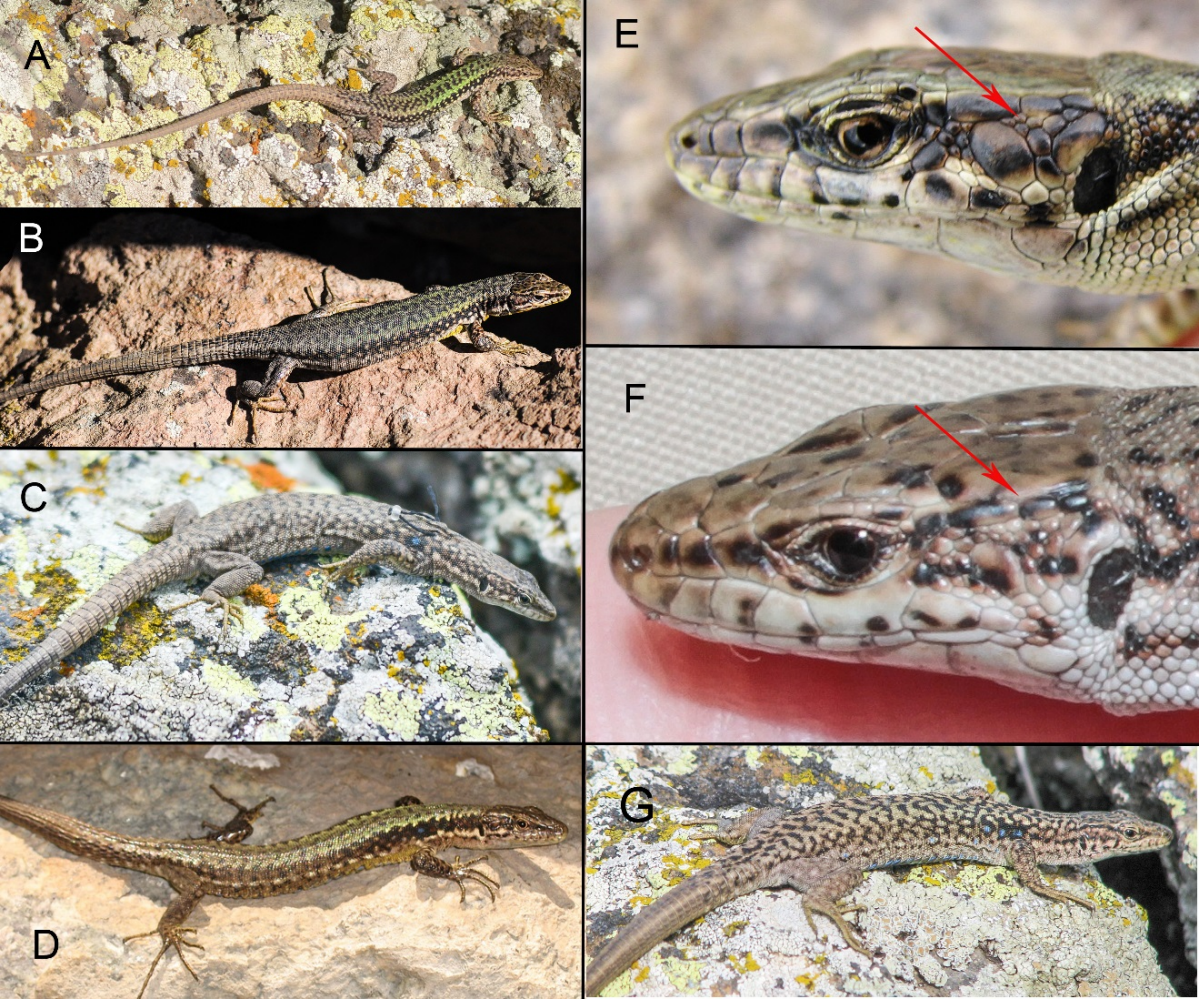
Morphology and external view of studied lizards A: Male *Darevskia valentini*; B: Female *D. valentini*; C: *D. unisexualis*; D: *D. armeniaca*; E: Head of *D. armeniaca* with one row of scales between tympanic shield and mid temporal scale; F: Head of *D. valentini* female; G: Female triploid hybrid between *D. valentini* and *D. unisexualis*.

Among rock lizards, Valentin’s rock lizard is a relatively large species with a snout-vent length (SVL) body size of up to 77 mm. According to Darevsky (1967, p. 104), the dorsum coloration is greenish-brown or bright-green and the venter is yellow or white (Figure 1A, B). A black temporal stripe bears one to three longitudinal rows of rounded, bright (bluish in pectoral zone) spots forming the centers of fused, dark ocelli. The upper side of the head has black, irregular blotches and spots. Males differ in body proportion, with a relatively wide head, and bright coloration. Daughter parthenogenetic species *D. unisexualis* differs from *D. valentini* by grey dorsum coloration with a reticulate pattern (Figure 1C). Unlike in *D. armeniaca* (Figure 1D), there are several, not one, rows of scales between the tympanic shield and mid temporal scale (Figure 1E, F; Darevsky, 1967, fig. 39, p. 52). *Darevskia armeniaca* differs from *D. valentini* by dull-green dorsal coloration with light spots along the flanks and pale-yellow venter with white throat (Figure 1D). The adult triploid hybrids between parthenogenetic *D. unisexualis* and *D. valentini* are often larger and more robust than the adult diploid individuals (Danielyan et al., 2008) and can be distinguished by green dorsum coloration with distinct reticulate pattern similar to that in *D. unisexualis* (Figure 1G). Hybrids between parthenogenetic *D. armeniaca* and *D. valentini* are larger than the diploids but exhibit no any obvious differences from *D. armeniaca*.

### Study area

**Figure 2:**
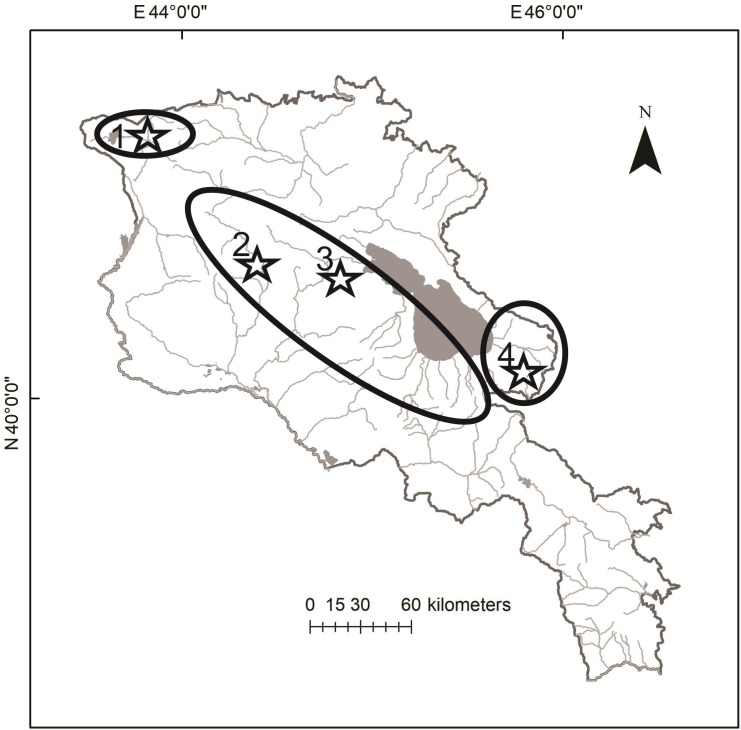
**Distribution map of Valentin**’**s lizard *(D. valentini*) in Armenia and adjacent areas (Arakelyan et al., 2011 with changes)** Survey areas are marked by stars, 1: Mets Sepasar (*D. valentini*; *D. raddei nairensis*; *D. armeniaca*), 2: Kuchak (*D. valentini*; *D. armeniaca*; *D. unisexualis*), 3: Lchashen (*D. valentini*; *D. armeniaca; D. unisexualis*), 4: Sotk populations (*D. valentini*; *D. armeniaca*).

**Figure 3:**
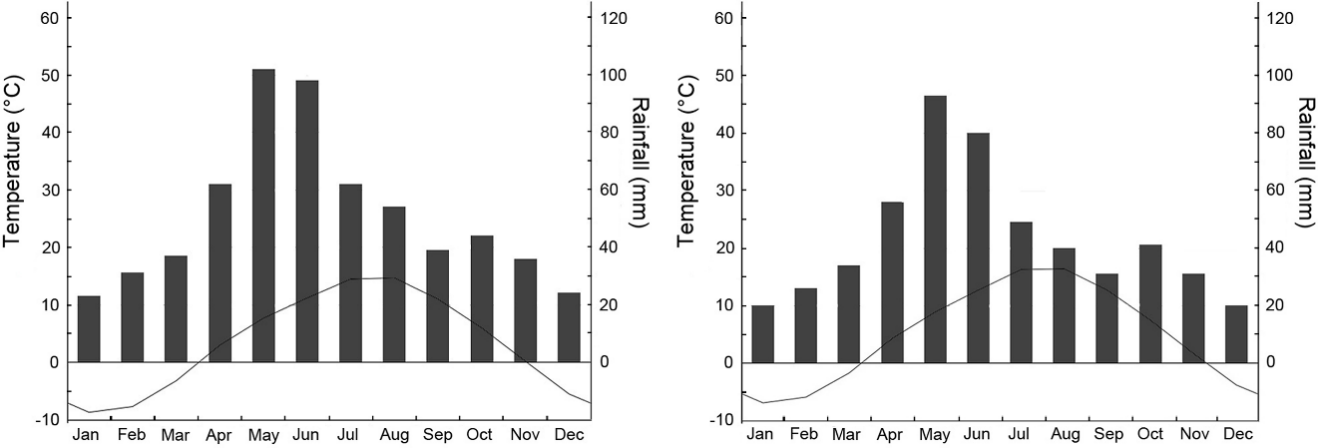
Climatographs (data from https://ru.climate-data.org) for cities closest to the main survey areas A: Ashotsk (Shirak Province, area of Mets Sepasar); B: Aparan (Aragatsotn Province, area of Kuchak).

**Figure 4:**
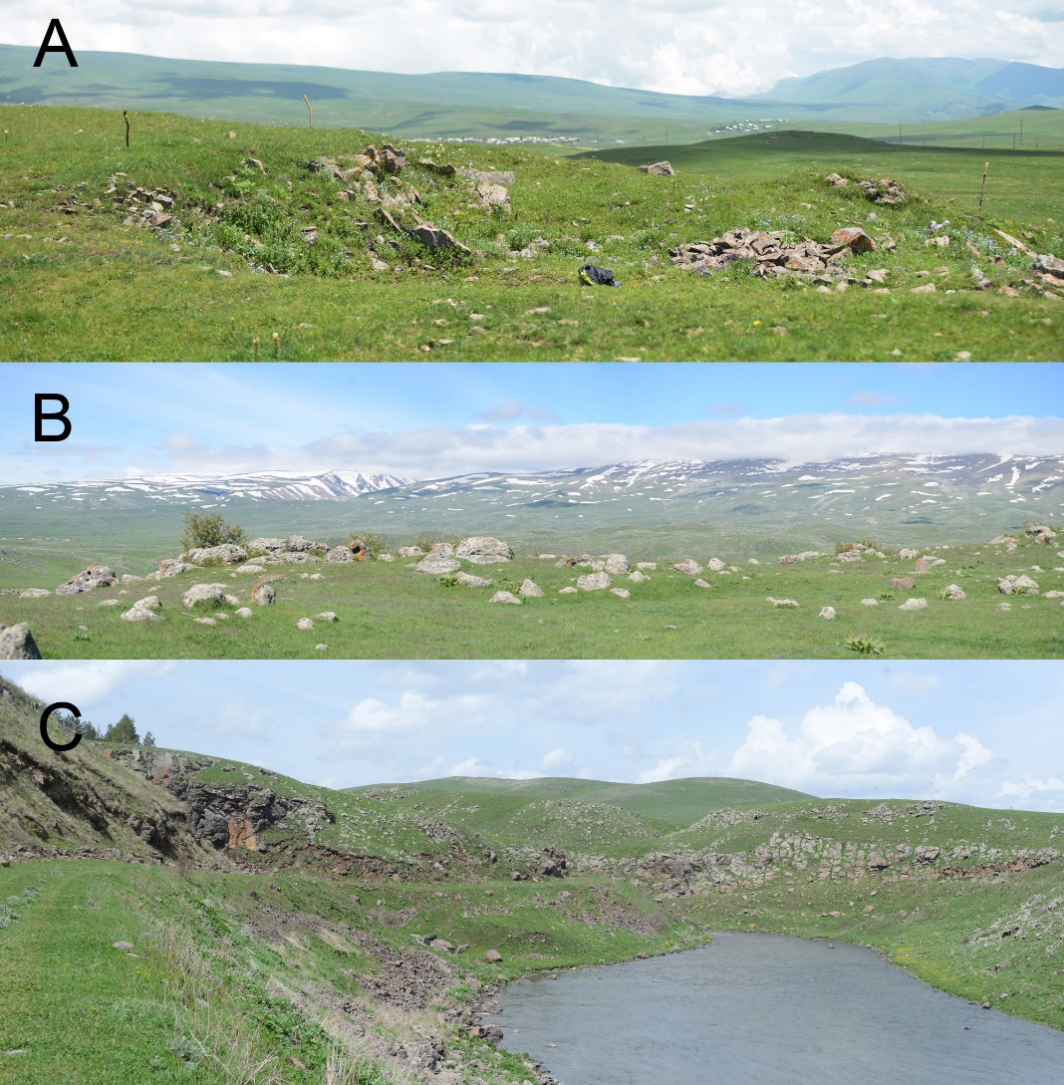
Typical landscapes inhabited by *D. valentini* High-elevation meadows with stony outcrops in Mets Sepasar (A) and Kuchak (B). Steep cliffs in the Akhuryan River valley in the Mets Sepasar area (C).

Valentin’s lizard is widely distributed along the Armenian highlands, mostly in the central and northern parts of the country. We used the data on distribution of *D. valentini* (Arakelyan et al., 2011; Petrosyan et al., 2019) and open access in ArcGIS 10.2 applying World Topographic Map, published by ESRI (services.arcgisonline.com) application to make the distribution map (Figure 2). We collected data for the present work between 2006 and 2017 from four locations in Armenia (Figure 2): Mets Sepasar (area of Ashotsk City, Shirak Province, 2 030 m a.s.l.), Kuchak (area of Aparan City, Aragatsotn Province, 1 900 m a.s.l.), Lchashen (Gegharkunik Province, 1 900 m a.s.l.), and Sotk (Gegharkunik Province, 2 030 m a.s.l.). Kuchak and Mets Sepasar were the main survey areas as they represent two population models with high and low population densities. In all studied localities, Valentin’s rock lizards occupy highlands with cold snowy winters, chilly and rainy springs and short, relatively hot and dry summers (Figure 3). Although the climatic conditions are similar in these areas, Mets Sepasar experiences heavier snowfalls in winter and, in general, colder temperatures. High-elevation meadows with dense grass vegetation of up to 40–50 cm in height dominate the landscape. In the survey areas, rock lizards inhabit stony mounds (Figure 4A), rocky outcrops of glacial origin, and steep rocks along roads (Figure 4B), riverbanks, and cliffs on lakeshores and river valleys. All listed population areas are subject to cattle grazing.

There are two population types: allopatric, where *D. valentini* is the only one species of rock lizards; and sympatric (Figure 2), where *D. valentini* coexists with the parthenogenetic Armenian rock lizard (*D. armeniaca* Meheli, 1909) and *D. unisexualis* (Darevsky, 1966).

### Population density and structure

We used distance sampling (Buckland et al., 1993) to estimate lizard population densities. Age and sex structure, microhabitat use, as well as seasonal and daily activities were estimated on the same line transects (Table 1). One line transect was placed in the high-elevation meadow area near Mets Sepasar (allopatric population) and another included the path along the bank of the Akhuryan River valley and where an old nineteenth century stone bridge crosses the river (sympatric population). The transect near Kuchak village crossed a meadow (sympatric population). Counting was performed by three scientists with the help of binoculars. All participants were experienced in joint work to calibrate their results. The perpendicular distance from the transect line to each detected lizard was noted in a field book. We visually identified the lizards to species and hybrids according to experience and described characteristics (Danielyan et al., 2008). We also noted age and sex categories: i.e., adult females, adult males, and young. The latter included subadults and juveniles due to difficulties in their discrimination. Prior to each survey, we recorded the temperatures above the surface in the shade and substrate temperatures in the sun. Encounter surveys were performed on selected transects from 0600 h to 1900 h during warm sunny days: one in Kuchak (23 June 2013) and two in Mets Sepasar (14 June 2016). This allowed us to estimate daily activity patterns of lizards and to define the maximum number of lizards per day for population density calculation.

**Table 1:** Data for population density and structure estimation

Area	Mets Sepasar (Allopatric)	Mets Sepasar (Sympatric)	Kuchak (Sympatric)
Survey dates	20., 21., 29. 05. 2016, 01., 14. 06. 2016	14. 06. 2016	17., 18., 23. 06. 2013
*N*_ad_ (*D. valentini*/*D*. spp)	317	320/98	15/783
*N*_y_	804	174	567
*l* (m)	800	1 000	1 000
*L* (m)	13 600	11 000	19 000
*w*	10	10	10
*P*a	0.779	0.318	0.253

*N*_ad_: Number of detected adult individuals; *N*_y_: Number of detected young animals; *l*: Transect length, *L*: Total length of all transects, *w*: Transect width, *P*a: Probability of underestimated individuals.

Population density (D) is commonly calculated as the number of detected animals (*n*) within the boundaries of the occupied area (A). We calculated population density via the formula D=*N*/2*lwPa*, where *l* is the length of the transect, *w* is half the width of the transect, *N* is the number of estimated lizards, and *Pa* is the proportion of animals detected in the covered area (2*lw*) (Kendeigh, 1944). The number of animals over substantial distances can be underestimated. To estimate the proportion of animals detected within the covered area, at different distances from the line of the transect, we divided the width of the transects into segments, which included perpendicular distances to each registered lizard: 0–1 m to 9–10 m. The detection function (df) for different distances from the counting transect was estimated, and the probability of underestimated individuals was calculated using the formula *Pa*=*S_df_*/*S_ed_*, where *S_df_* is the area under the detection function and *S_ed_* is the area under uniform distribution within the detection range (Buckland et al., 1993; Gonzalez et al., 2017). Detection functions were determined during daylong monitoring and fitted to half-normal distribution (Table 1; Supplementary Figure 1). The differences in *Pa* between the study areas were explained by low temperatures and strong winds in the meadow near Mets Sepasar village; hence, lizard activity was higher on the stones, where the probability of being detected by an observer was also higher. Further recalculations to the area A were established, which is, in our case, equal to one hectare. Calculations were performed using the “Distance” package in R software. Average lizard density was calculated for the samplings at activity peaks and is given with the standard errors (*SE*) for each line transect. Average population density (±*SE*) was calculated from the activity peaks (the highest number of lizards registered on the transect during the whole-day registrations). Although the distance sampling method might underestimate actual population density (Smolensky & Fitzgerald, 2010), it is important for describing seasonal and daily activity both in terms of age and sex structure of the population. A similar method has been used in previous studies on *Darevskia* (Galoyan, 2010; Tsellarius & Tsellarius, 2001), facilitating comparison with our data.

Local population density was also estimated by the capture-mark-recapture method (CMR) in the sampling areas in Kuchak (3 600 m^2^) and Mets Sepasar (1 300 m^2^). Lizards were captured and individually marked by amputation of the distal phalanx and by two color beads sewn by a nylon line through the skin fold on their back (Fisher & Muth, 1989). The latter method allows observing and distinguishing individuals from a distance. These two humane methods have no negative impact on lizard behavior (Galoyan, 2017; Husak & Fox, 2003; Nicholson & Richards, 2011; Tsellarius & Tsellarius, 2001).

In total, 118 lizards were marked in 2013 in the study area near Kuchak village (92 *D. armeniaca*, six possible *D. armeniaca×D. valentini* hybrids, 11 *D. unisexualis*, six *D. unisexualis×D. valentini* hybrids, and three *D. valentini* males) and in 2016, 176 lizards were marked within the study area near Mets Sepasar (*D. valentini*, allopatric population). Most lizards were marked in the first days after emergence from winter shelters, with newcomers marked if they occurred in the study area afterwards. For each individual, we measured body length with a caliper to an accuracy of 0.5 mm; photographs were taken for further identification of the specific coloration pattern; for skeletochronological studies, the removed finger was measured and dried. To estimate the population density in the sampling areas, we used the Petersen-Lincoln index (Southwood & Henderson, 2000) using the formula *N*=*Kn*/*k*, where *N* is the number of animals in the population, *K* is the number of animals captured the second time, *n* is the number of animals marked the first time, and *k* is the number of recaptured animals marked. Population density was estimated for seven pairs of full observation days (26 May–5 June 2016) in Mets Sepasar and for five pairs of full observation days (23 May–5 June 2016) in Kuchak.

### Age determination

The lizards oviposit in late June–August (Darevsky, 1967). The first wintering of juvenile rock lizards occurs when SVL is about 30–40 mm (Arakelyan & Danielyan, 2000; Tsellarius & Tsellarius, 2009). This was supported in the present work for *D. valentini* by skeletochronological age estimation for four individuals (32–36 mm). The age of young animals (1–3 years old) was partly based on SVL measurements; lizards with SVL=32.4±0.96 (*n*=9, May–June) were considered juveniles (juv). Subadults (sad) were defined as those lizards which survived two and three winters and showed an SVL of up to 60 mm. Individuals with an SVL higher than 60 mm and which survived more than four winters were defined as adults. Age estimations of 65 older individuals from Mets Sepasar, 22 from Lchashen, 10 from Kuchak, and 15 from Sotk were based on the skeletochronological method (Castanet, 1994; Smirina, 1974). The amputated digits were prepared and analysed following standard methods (Galoyan, 2013a; Kurnaz et al., 2017; Tsellarius & Tsellarius, 2009). The phalanxes were decalcified in 5% nitric acid and microscopic sections (10–15 µm) were prepared on a sledge microtome MC-2. The sections were colored by Ehrlich’s hematoxylin.

### Space use

Visual observations of individually marked lizards took place from 15 May–14 June 2014, 13 May–15 June 2016, and 25 September 2016 in Mets Sepasar and 21 May–22 July 2013 in Kuchak. Each sampling area was mapped in detail. Visual observations of active lizards were performed between 0800 h and 1800 h on days with favorable weather. Focal observations were made from 5–10 m or closer, especially if the lizards were habituated to the presence of an observer. The observer chose a focal lizard and observed it for 10–15 min before switching to another individual. Location, contacts, including mating and mating attempts, and foraging behavior were recorded using a Nikon D600. Total observation time was 30 d (143 h) in 2013 in Kuchak and 17 d (49.5 h) in 2014 and 19 d (65.5+78.5 h) in 2016 in Mets Sepasar. The position of the focal animal was noted each minute and converted into location points for range estimation.

Here, we defined home range as the minimum convex polygon (MCP), with the exclusion of occasional sallies beyond the home range and characterized by specific behavior (Tsellarius & Tsellarius, 2005). Range structure was defined for residents by approximately 300 location points, which included at least 75% of the range area (Galoyan, 2013a; 2017).

### Seasonal activity and reproduction cycle

During the visual observations, we noted and described successful copulation and mating attempts. We considered the mating season as the period between the first mating attempts and the appearance of females with obvious marks on their bellies (i.e., jaw bite traces) and last observed mating attempts. The oviposition period was defined as the time between the appearance of the first and last females who laid eggs within the study area. The skin fold along the body flank, just after clutching, was used to distinguish oviposited females. Fourteen females were caught within one week before egg laying and kept in a terrarium. The number of eggs per clutch was counted and egg size parameters were measured with a caliper. The eggs and females were returned to the population after the study.

### Feeding

We used two methods to estimate prey item composition and proportion: visual observations and contents of feces collected in the field. The observations were recorded with a camera in video mode and prey items were determined from the images. The prey items from the feces were determined under a binocular after fecal maceration in warm water. In total, 54 fecal samples, which included 205 prey items, and 32 records were analysed.

We also estimated prey density in the study area. Total counts of invertebrates in grass, on substrate surfaces, and in shallow surface areas were performed within a random 25 cm×25 cm plot (*n*=19) (Tsellarius & Tsellarius, 2001). The numbers of feeding objects were counted on a cold day (19 June) in Mets Sepasar, when flying insects were immobile on the surface and could easily be detected in the grass.

### Thermal biology

Body temperature (*T*_b_) was measured from 3–15 June 2016 in Mets Sepasar and from 15–20 June 2015 in Kuchak. We measured active lizards captured in the field (41 records in Mets Sepasar and 23 in Kuchak) by inserting a cloacal thermometer (Miller-Weber cloacal thermometer). Subsequently, 10 females and 10 males from Mets Sepasar were brought to the laboratory to estimate the thermal preferences of lizards. Lizards were acclimated for 3 d prior to the experiment. In the laboratory, we created a thermal gradient ranging from 15–53 °C in an indoor 100 cm×50 cm×50 cm glass terrarium by suspending light bulbs and ice blocks. Every hour from 0800 h to 2000 h, we measured the body temperature of the lizards and ambient temperature using a Miller-Weber cloacal thermometer.

### Statistical analyses

All means are given with standard errors (*SE*). We used the independent sample *t*-test to compare range areas between males and females after applying the Shapiro-Wilk normality test for choosing criteria (parametric or nonparametric). We used the Wilcoxon test for comparison of the SVL of males and females of the same age and body temperature of males and females from the same and different populations. Spearman rank correlation (*R*s) was calculated to estimate the correlation between age and SVL of lizards, body temperatures and ambient temperatures, and body temperatures and substrate temperatures.

## RESULTS

### Population density

The average population density of the Valentin’s lizard varied in different locations from 3 to 66 ind./ha (DSM census). Local density within a sample site (CMR census) reached up to 400 ind./ha (Table 2). This number was comparable with the population density of parthenogenetic species in Kuchak (*D. armeniaca* and *D. unisexualis* and their hybrids with *D. valentini*), where Valentin’s lizard coexists.

**Table 2:** Average (±*SE*) population density of adult lizards, calculated by the distance-sampling method (DSM), on the line transects at activity peaks in three populations

	Elevation (m)	Biotope	*D. valentini *(ind./ha)		*D. *spp. (ind./ha)	
Method			DSM	CMR	DSM	CMR
Mets Sepasar (Allopatric)	2 030	Meadow	25.7±1.60	433.9±28.40	–	–
Mets Sepasar (Sympatric, Akhuryan gorge)	2 030	Gorge, river valley	66.0±6.55	No data	18.9±1.57	No data
Kuchak (Sympatric)	1 900	Meadow	3.0±0.99	5.6±0.79	159.6±6.06	194.4±7.04

Mets Sepasar allopatric; Mets Sepasar sympatric; Kuchak sympatric: (*L*=4 800, 3 000, 4 000 m; *N*=192, 162, 329 ind.; *P*a=0.779, 0.318, 0.253, respectively, where *L*: total route distance on the transects; *N*: number of detected animals; *P*a: “average” detectability of animals on route). Local average (±*SE*) population density at studied settlements determined by capture-mark-recapture method (CMR) in two locations: Mets Sepasar (A=1 300 m^2^) allopatric and Kuchak (A=3 600 m^2^) sympatric. A: Correspondingly, where A is the sampling area. –: Not available.

The ratio of *D. valentini* to other species of rock lizards differed among sites (Tables 2, 3). For instance, *D. valentini* was twice as common as *D. armeniaca* in the Akhuryan River gorge, although in the Kuchak population, individuals in the biparental species were extremely scarce (Tables 2, 3).

### Population structure

The male/female proportion in *D. valentini* was close to 1:2 in the allopatric population and about 1:1 in the sympatric population in Mets Sepasar. If we included the parthenogenetic *D. armeniaca*, the male to female proportion was the same (Table 3). The sex ratio was dramatically biased in Kuchak, where the number of *D. valentini* females was five times lower than that of males, with males compensating for their absence by interactions with females of parthenogenetic *D. unisexualis* and *D. armeniaca*.

**Table 3:** Proportions of males and females of biparental species *D. valentini*, parthenogenetic *D. armeniaca*, and young animals (Sad) on the transects on 14 June 2016 in Mets Sepasar and 23 June 2013 in Kuchak

Transect	*D. valentini* F (%)	*D. valentini* M (%)	*D. *spp. Ad (%)	*D.* spp. + *D. valentini* Sad (%)	Length of route per transect (m)	*n*	*N*
Mets Sepasar allopatric	18.1±2.21	10.4±1.45	–	71.5±5.19	1 900	16	1 096
Mets Sepasar sympatric	21.6±1.92	24.5±1.56	23.2±3.27	30.8±4.70	1 000	11	494
Kuchak sympatric	0.2±0.16	1.0±0.32	58.8±2.15	40.0±2.23	1 900	19	1 369

*n*: Number of routes per transect, *N*: Number of registered lizards. M: Male; F: Female. Ad: Adult; Sad: Subadult.

The proportion of adults to juveniles was considerably biased to the young in the Mets Sepasar meadow (Table 3) and, albeit to a lesser extent, in the Akhuryan River valley and the sympatric population in Kuchak.

### Age and body length

The oldest lizards were one male and one female, which survived at least nine winter hibernations. Only a few males that survived more than six winters were found, which suggests that, on average, females reach higher ages than males (Table 4).

**Table 4:** Mean (±*SE*) body length (mm) values of male and female *D. valentini* from Kuchak, Lchashen, Mets Sepasar, and Sotk; ages defined by skeletochronological analysis

	Juv 1	Sad 2	Sad 3	Ad 4	Ad 5	Ad 6	Ad 7	Ad 8	Ad 9	
	Winter	Winters	Winters	Winters	Winters	Winters	Winters	Winters	Winters	
M	32.4±1.44	53.5±0.40	58.2±1.05	64.4±0.96	66.2±0.56	67.4±0.87	No data	69	68	
F		48.6±1.58	58.0±1.76	62.1±1.50	70.2±0.74	71.7±1.06	73.6±1.35	No data	76	
*N*	4	3M+8F	7M+5F	11M+10F	17M+16F	5M+11F	0M+9F	1M+0F	1M+ 1F	

*N*: Number of individuals. M: Males; F: Females. Juv: Juvenile; Sab: Subadult; Ad: Adult.

**Figure 5:**
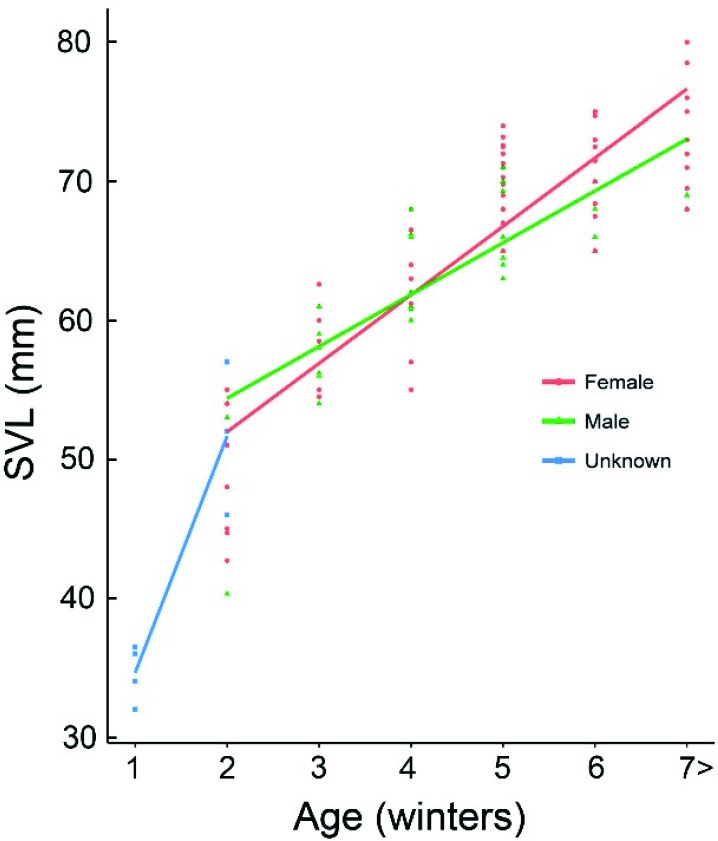
Body lengths and ages of lizards from four populations (Mets Sepasar, Sotk, Lchashen, and Kuchak) *n*=112 ind. There was a positive correlation between age and SVL for males and females (*R*s=0.740, *P*<0.0001 in males and *R*s=0.825, *P*<0.0001 in females).

The maximum SVL was 79 mm (a female from the Mets Sepasar population). The growth rates of males and females in Mets Sepasar were almost the same before the fourth winter (Wilcoxon test, *P*>0.05); however, male growth slowed after the fifth wintering, whereas females continued to grow (Figure 5, Table 4) and became significantly larger than males of the same age (Figure 5, W=16, *P*=0.04 and W=3.5, *P*=0.03, respectively, for five and six winter hibernations).

### Space use

Lizards were concentrated in areas with stony mounds and rocky outcrops on the highland meadows in both Kuchak and Mets Sepasar. The highest concentration of lizards was observed around winter shelters, where the local density of lizards (CMR) was significantly higher in the observation areas compared to the average density on the transect at the same area (Table 2).

Not all lizards observed within the survey areas lived continuously within the same range. The number of residential individuals (observed during the entire observation period) in the Mets Sepasar allopatric population was 32 (19% of all captured animals in 2016). Among the 148 lizards captured in Kuchak in 2013, only three residential *D. valentini* males (2%) were observed.

The home ranges of Valentin’s lizards consisted of stony mounds and solitary stones, which were used as shelters and basking sites. Two types of individual home ranges were detected: monolite (basking sites close to each other and connected by permanent routes, one core area) and fragmented (basking sites far from each other and not connected by permanent routes, more than one core area). Residential individuals could use both of types of home ranges. Fragmented home ranges (121.1±12.38 m^2^) were larger for males than monolite home ranges (46.6±5.56 m^2^) (*t*=5.49; *P*=0.0003, *n*_1_=8, *n*_2_=17), and male monolite home ranges were larger than that of females (22.1±4.56 m^2^) (*t*=3.41; *P*=3.4074; *P*=0.002; *n*_1_=17, *n*_2_=14). Several females jointly used an area and often basked on the same stones simultaneously, sometimes with one male. Males were aggressive towards each other; however, their ranges could also overlap. Some males seemed to have harems comprising residential and switching females. Males in the Kuchak population also had social and sexual interactions with *D. armeniaca* and *D. unisexualis* females.

### Seasonal and daily activity

Emergence of *D. valentini* in the area of Mets Sepasar after winter hibernation was observed from 15 May in 2014 and from 12 May in 2016. Spring activity in the morning started at about 1000 h, when the ambient temperature reached 10 °С and the shelter temperature was 7 °С. The sex ratio just after leaving the winter shelters was 1:1. Due to permanent rains and cloudy weather in May in Kuchak, mass emergence from the winter shelter in 2013 appeared on 21 and 22 May, when ambient air temperature was 11 °С, whereas in 2016, the first appearance of lizards was detected on 20 April, with massive emergence in the first days of May.

**Figure 6:**
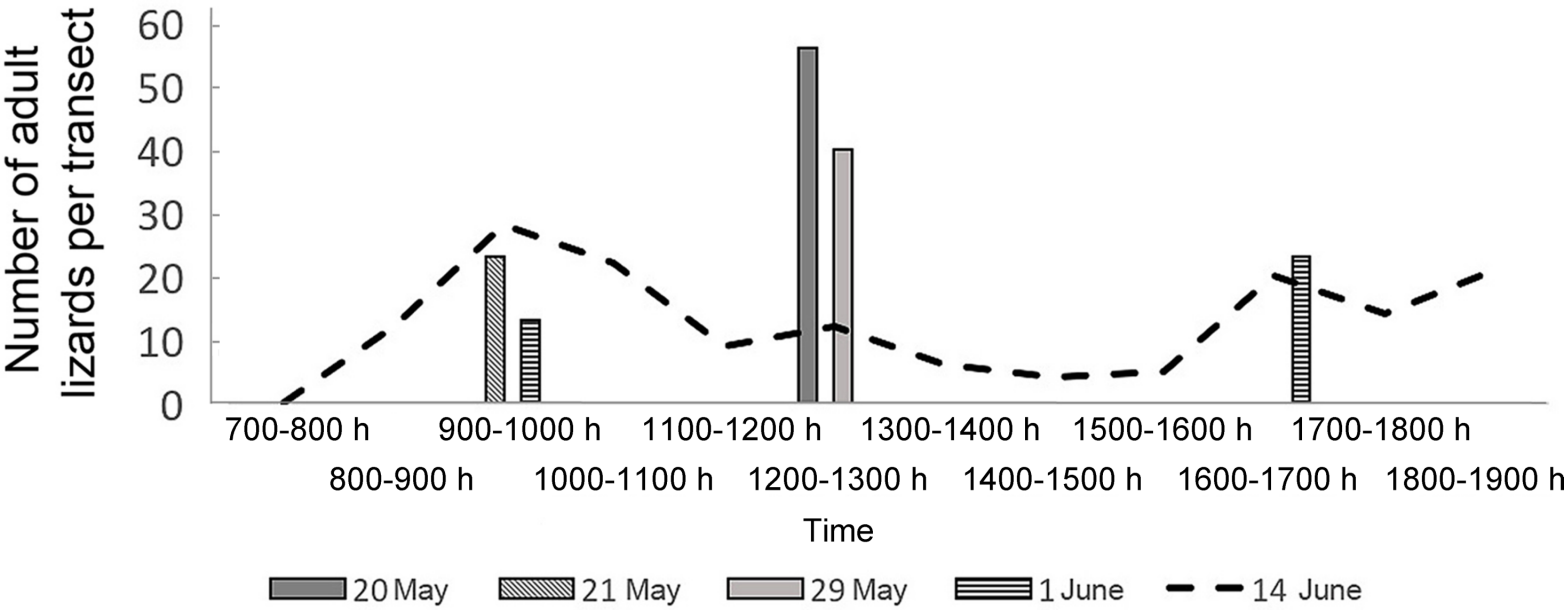
Number of observed adult lizards on the transect in Mets Sepasar on the high-elevation meadow in 2016

Lizard activity in late May and June showed two peaks (morning and evening) (Figure 6). Due to the low ambient temperatures, lizards basked and spent more time on the surface and were thus more visible in May than in the summertime; hence, the maximum number of lizards per one route on the transect on the transect was higher.

**Figure 7:**
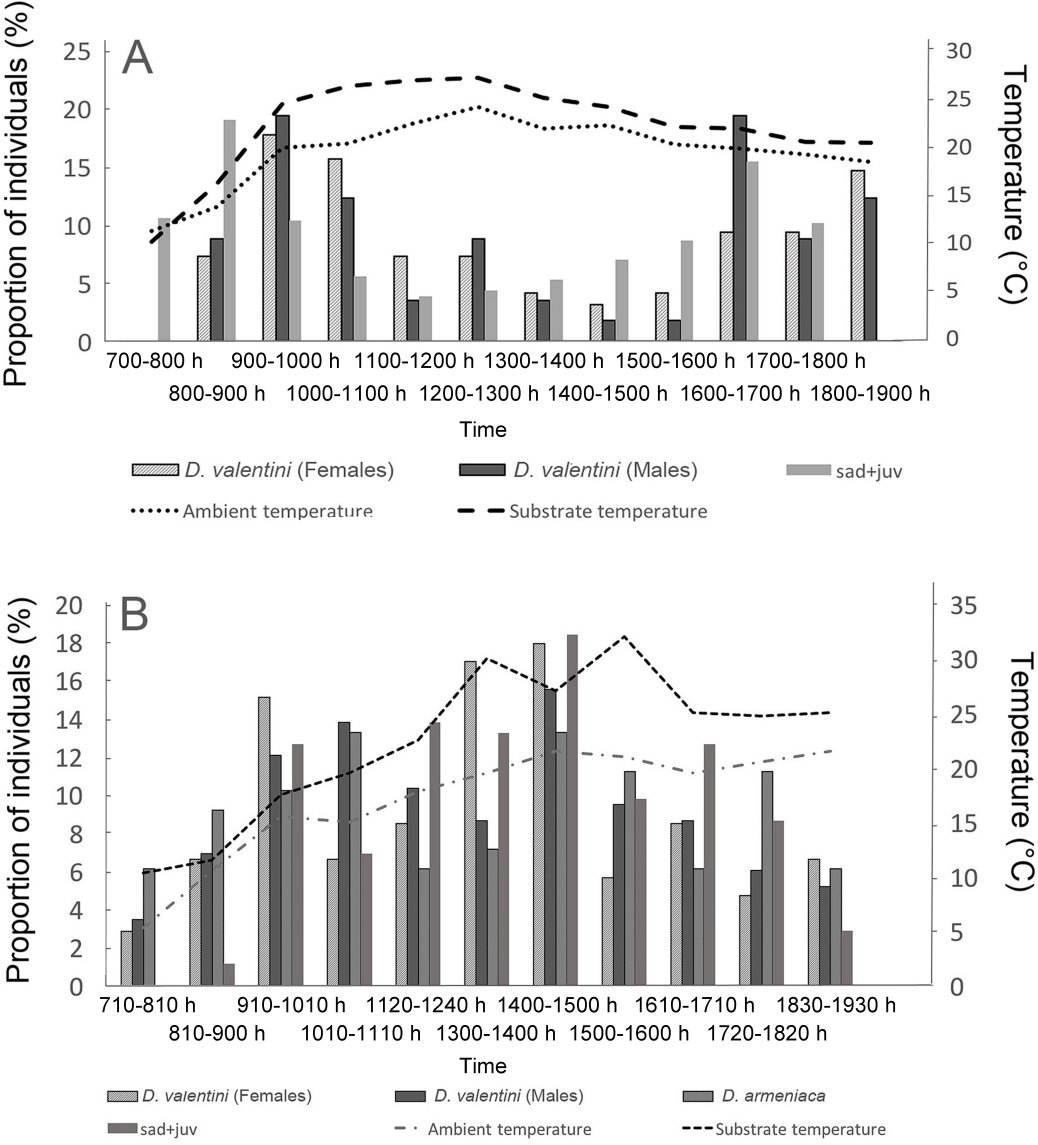
Daily activity of *D. valentini* in Mets Sepasar on the high-elevation meadow (A) and Akhuryan River valley (B) on 14 June 2016 Juveniles and subadults of these two species look similar from a distance and were therefore combined.

In late May to early July, air temperatures increased, and daily activity started at 0800 h, though some individuals could be observed as early as 0700 h (Figures 5; 6A, B). In spring, lizards entered shelters at about 1700 h, whereas in mid-summer, they entered shelters between 1900 h to 2000 h. On 25 September 2016, lizards were still recorded on the surface and were mainly found basking near their winter shelters.

### Reproductive cycles and intersexual behavior

The mating period of *D. valentini* started just after leaving the hibernation shelters at the beginning of April and lasted until early June. We recorded three mating attempts in 2014 between 19 and 31 May and 10 mating attempts between 19 and 27 May in 2016 in the Mets Sepasar population. In the Kuchak population, with the low density of female *D.valentini*, we observed 11 mating attempts of *D.valentini* males with parthenogenetic females of *D. armeniaca* and *D. unisexualis* from 21 May to 9 June 2013.

**Figure 8:**
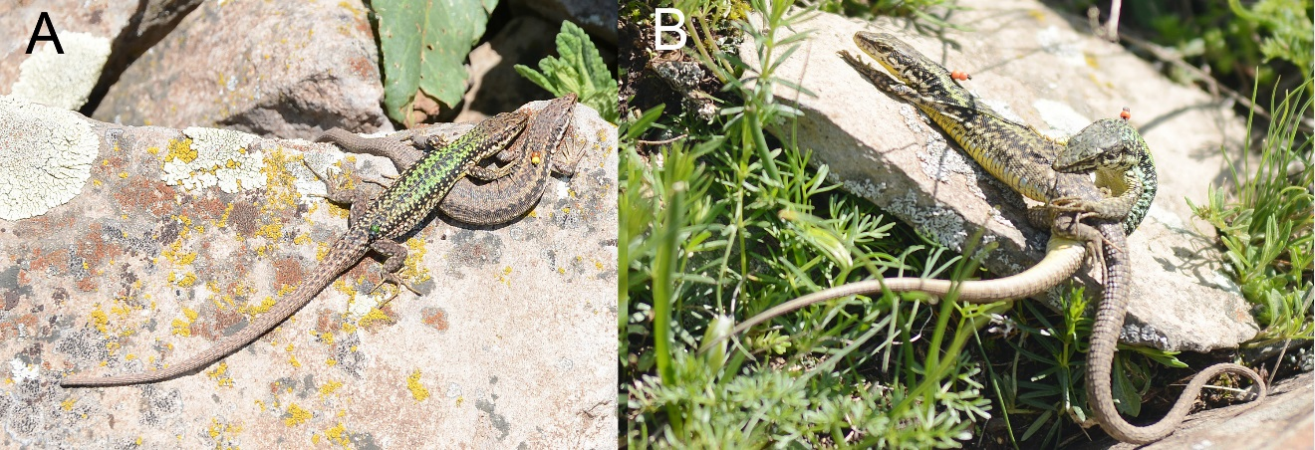
Joint basking (A) of male N149 and female N140 in 2016; mating (B) of male N8 and female N13 in 2014

During the mating period, the male and female often basked together, with physical contact: i.e., a male could lay on a female or vice versa, and they often crawled over each other (Figure 8A). This kind of behavior was considered as social (affiliative). Males could switch from this activity to sexual behavior. We also observed other types of sexual behavior, including a male chasing a female and attempting to mate without preliminary social contact (sexual aggression). During coitus, the male took the female’s lateral fold in his jaws and coiled over her, restraining the female by hugging her in the crest area via his hind limbs (Figure 8B). The intraspecies coitus lasted on average 19.9±3.33 min (*n*=7), with a maximum duration of 37 min. Mating attempts, even unsuccessful ones, resulted in jaw-prints on the flanks of the female. These marks were used as an indicator of such an attempt but did not necessarily indicate successful mating. Larger females with a SVL > 65 mm mated earlier than smaller individuals.

Females oviposited from 14 to 29 June 2016 (*n*=10) in the Mets Sepasar population. Pregnancy in Valentin’s lizard lasted 45–50 d. Not all adult females laid eggs: three (SVL=65, 67, and 70 mm) were observed to mate within the study area in the Mets Sepasar population, but did not oviposit. The average number of eggs per clutch in adult females older than three years and larger than 60 mm of SVL was 6.0±0.50 (*n*=26). The maximum number of eggs per clutch was greater in larger and older females, but this number was not strongly correlated with the SVL of females (*R*s=0.43, *P*=0.027, *n*=26), (Table 5). Mean egg size was 14.8±0.23 mm×8.1±0.13 mm (*n*=33).

**Table 5:** **Snout-vent length (SVL) and number of eggs per clutch of Valentin**’**s lizards from the Mets Sepasar population**

Age	3	4	5	6	> 7
SVL (mm)	57.5	63.0±2.73	70.6±1.01	72.4±1.73	73, 71
Number of eggs per clutch	2	5.5±1.04	6.1±0.64	7.8±2.17	6, 2
Number of females	1	4	8	4	2

Age was estimated via the skeletochronological method.

### Feeding

**Figure 9:**
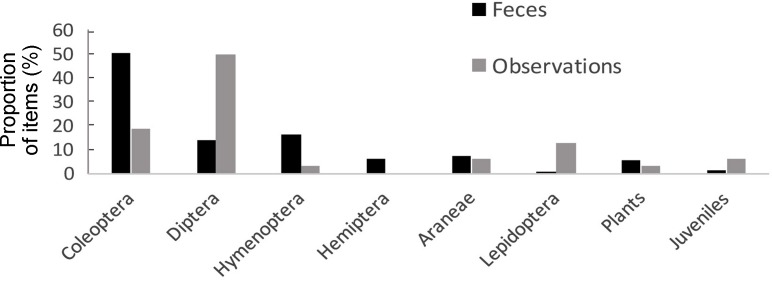
**Distribution of feeding objects found in feces of Valentin**’**s lizards (*n*=205) and by observation (*n*=32)**

**Figure 10:**
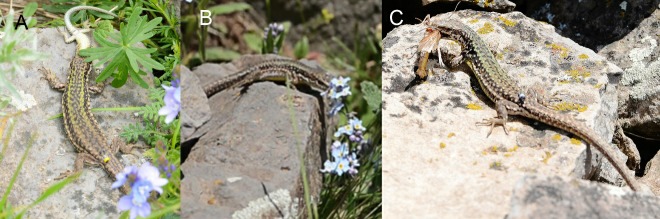
**Feeding observations of the Valentin**’**s rock lizards** А: Adult female eating a juvenile; B: Subadult eating“forget-me-not”; C: Female catching a locust.

While Valentin’s rock lizards are primarily insectivores, we did observe consumption of a small amount of vegetation (e.g., plants, flowers) in the current study (Figures 9, 10). The abundance of possible prey was relatively high in the Mets Sepasar population (mean number of feeding objects 4.5±0.69, *n*=18 samples of 25 cm×25 cm frames). According to the feeding objects found in feces, *D. valentini* mainly fed on beetles (Coleoptera). Direct observations indicated that flies (Diptera) were also a favorable food, which they hunted from the stems of plants or in flight (Figure 10), with Hymenoptera, Lepidoptera, and Aranea consumed rarely. During our observations, two cases of infanticide were observed (one male and one female ate a juvenile). We also recorded remains of juveniles in feces (Figures 8, 9).

The lizards rarely actively searched for prey outside their main activity areas. Most often, they preyed within and near basking sites or on the move (during cruising foraging, *sensu* Regal, 1983). We recorded hunting behaviour (e.g., chasing and ambushing) exhibited in adults preying on juveniles.

### Thermal biology

No significant differences between the body temperatures of adult lizards (within and between sexes) were revealed in the Kuchak and Mets Sepasar populations during the study periods (Wilcoxon test, *P*>0.05), although substrate temperatures were higher in the Mets Sepasar allopatric population (Table 6).

**Table 6:** Average (±*SE*) body temperature (*T*_b_) of active adult male and female *D. valentini* from Kuchak and Mets Sepasar, determined via a thermo-gradient apparatus

	Male *T*_b_ (°С)	Female *T*_b_ (°С)	Substrate *T* (°С)	Ambient *T* (°С)
Kuchak	31.8±0.44 (*n*=19)	32.7±0.76 (*n*=4)	27.6±1.00	26.7±1.09
Mets Sepasar	31.7±0.90 (*n*=8)	32.3±0.53 (*n*=21)	32.0±1.23	23.0±0.56
Thermal gradient apparatus	31.5±0.39 (*n*=10)	31.8±0.59 (*n*=9)	32.4±0.60	32.4±0.60

*n*: Number of individuals.

**Figure 11:**
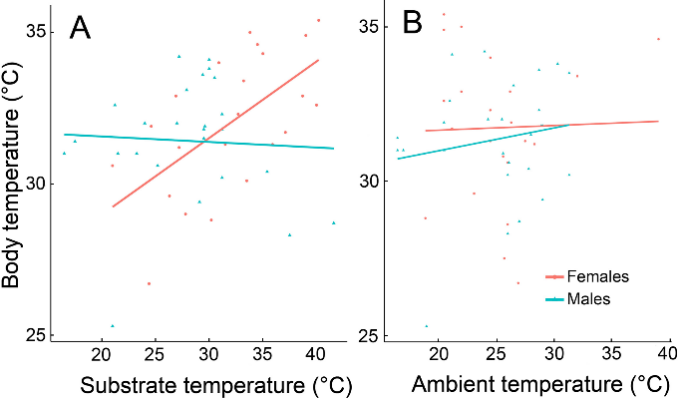
Lizard body, substrate and ambient temperatures A: Measurements of body temperature of male and female *D. valentini* from Sepasar and Kuchak (*n*=25F+27M). There was a positive correlation between female body temperature and substrate temperature (*R*s=0.644, *P*=0.002), but not between male body temperature and substrate temperature (*R*s=–0.0256, *P*=0.90. B: No relationship was found between body temperature and ambient temperature (Males: *R*s=0.0778, *P*=0.70; Females: *R*s=0.1208, *P*=0.38).

Body temperatures of adult lizards that had just left their shelters were almost the same as the ambient temperatures at the capture site (*Т*_b_=28.5±3.07 °С and *T*air=25.3±2.18 °С, *n*=4, respectively). Hence, basking is an obligate activity for Valentin’s lizards, enabling them to reach higher temperatures to perform activities (*T*_b_=31–32 °С, Table 6). Lizards came out of their shelters and flattened themselves on a stone, exposing the maximum amount of their body surface to the sun. They could also obtain heat from the stones when the sun disappeared behind clouds. Thus, lizards managed to maintain a high body temperature even when air temperatures were 14–16 °C or less. Lizards switched to shuttling behavior (Spellerberg, 1972) during hot days and moved from the shade to sunny spots to maintain stable temperatures. Activity decreased with increasing temperatures in July; lizards were almost absent on the surface after 1400 h when the substrate temperature approached 30 °C (Figure 11).

Unlike females, males demonstrated no correlation between substrate temperature and body temperature (Figure 11). This was also the case for ambient temperature and body temperature.

**Figure 12:**
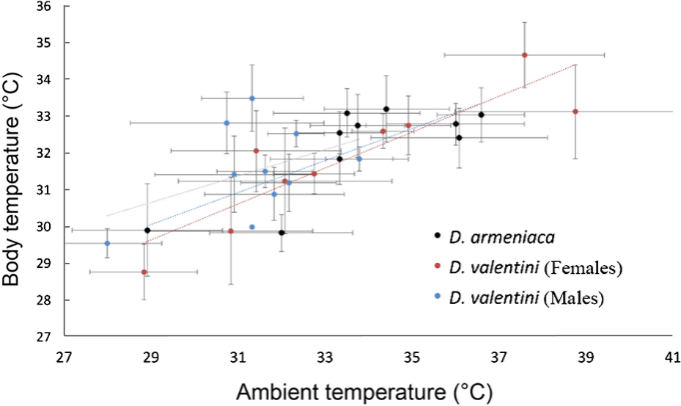
Mean (±*SE*) body and preferred ambient temperature of female parthenogenetic *D. armeniaca* (*n*=10), female *D. valentini* (*n*=9), and male *D. valentini* (*n*=10) from Mets Sepasar Each individual was measured 12 times throughout the day.

We found no difference between the body temperatures of active male and female Valentin’s lizards measured in nature and in the thermogradient apparatus (Wilcoxon test, *P*>0.05). Several females of parthenogenetic *D. armeniaca* and gonochoristic *D. valentini* preferred slightly higher ambient and substrate temperatures in the thermogradient apparatus than males of *D. valentini* (Figure 12), although no significant differences were revealed between females of these two species or between females and males of Valentin’s lizard (Wilcoxon test, *P*>0.05, Figure 12).

## DISCUSSION

The population density of *D. valentini* varied greatly from relatively low (1–2 ind./ha) to several hundred ind./ha, which exceeded the density of other biparental species such as *D. brauneri* (25 ind./ha, DSM, Tsellarius & Tsellarius, 2001) and *D. portschinskii* (5–6 ind./ha, DSM, Galoyan, 2010; Trofimov, 1981) (Table 2). Populations of parthenogenetic species *D. armeniaca* and *D. unisexualis* have also been reported to reach high densities (100–300 ind./ha, DSM, Galoyan, 2010; Tarkhnishvili et al., 2010; Trofimov, 1981; CMR, Danielyan, 1971). The possible explanation for the relatively high population density of *D. valentini* could be joint space use, as observed in parthenogenetic rock lizards (Galoyan, 2013b; Trofimov, 1981). The high population density with a high proportion of juveniles indicated good breeding and feeding conditions in Mets Sepasar. However, such a high population density may also have disadvantages. We suggest that the considerable number of lizards in a relatively small area may be why some females did not lay eggs, even after mating. Pregnancy termination, known as the Bruce effects, has been described in rodents (Blumstein, 2000) and may also occur in rock lizards. Another consequence of the large number of animals and high proportion of juveniles was infant cannibalism (Figures 9, 10). Infanticide has been observed for a variety of reptilian taxa (Ataev, 1985; Bogdanov, 1965; Jenssen et al., 1989), including rock lizards (Tsellarius et al., 2008). In some reptiles, eating unrelated juveniles of the same species may increase parental protection and may even be a possible reason for social evolution towards nuclear family formation (O’Connor & Shine, 2004). There are no studies devoted to this phenomenon in reptiles; however, a long-term study on barn swallows (*Hirundo rustica*) demonstrated decreased infanticide in a decreasing population (Møller, 2004).

Interspecies competition for resources can decrease the population size of competitors (MacArthur, 1958; Pianka, 1973; Schoener, 1971). Due to a faster reproduction rate, parthenogenetic species of rock lizards are likely better competitors, which should outrange related biparental species in overlapping areas in number (Tarkhnishvili et al., 2010). In some sympatric populations, the number of parthenogenetic rock lizards far exceeds the number of biparental relatives, as observed in Kuchak (Danielyan et al., 2008; unpublished data). However, this was not the rule in our study: the ratio of parthenogenetic and biparental species in the Akhuryan River valley was about 1:2, whereas the ratio of parthenogenetic Armenian lizards to female Valentin’s lizards was 1:1 (Table 3). In Sotk and Lchashen, *D. valentini* was also a common species (Figure 2). In our surveys on 23 June 2010 and 1 July 2010 in Sotk, we detected 19 adult *D. valentini* among 27 captured lizards of other rock lizards, whereas in Lchashen, we observed 24 Valentin’s lizards among 32 other individuals of the genus *Darevskia*. The deprivation of females belonging to biparental species in Kuchak may lead to regular hybridization between male Valentin’s lizards and parthenogens, whose proportions have exceeded 50% in some years (Arakelyan et al., 2011; Danielyan et al., 2008). No *D. armeniaca*×*D. valentini* hybrids were observed in the Akhuryan River gorge, and they were also extremely rare in Lchashen. Contrary to the Mets Sepasar population, for which a high density of females and males of *D. valentini* was described, in the Kuchak population, we revealed other interactions and social behaviors between *D. valentini* males and other parthenogenetic lizards (*D. armeniaca* and *D. unisexualis*) as a result of a deficit in females of their own species. Therefore, the three different species in Kuchak function as single population, where males are represented by *D. valentini* and females are represented by parthenogenetic species *D. armeniaca* and *D. unisexualis*. Moreover, in the Akhurian population where *D. valentini* coexisted with only one parthenogenetic species, we did not detect any interspecific hybrids, which is evidence that Valentin’s males in an area with a high density of females of their own species do not mate with other females of another species.

The estimated home range size of the Valentin’s rock lizard was comparable with the home range size in other species of rock lizards (Galoyan, 2013a; Tsellarius & Tsellarius, 2005). As in other saurian species, the home range of males was larger than that of females (Perry & Garland, 2002). The average home range of residential individuals was only about 20–40 m^2^, though some males had very large ranges (more than 100 m^2^). We suggest that females aggregating around small areas filled with stones, where they can find shelters and suitable basking sites, may explain the relatively small individual areas in males. Hence, males concentrated around these areas to maintain close proximity to females. This hypothesis should be further evaluated, although it might be supported by the knowledge that space use in females is more dependent on resource availability and distribution, whereas the behavior of males is more ‘female-orientated’ (Baird et al., 2003; Davies, 1992; Stamps, 1977; Tsellarius et al., 2017). Hence, small and overlapping home ranges favor high population densities and facilitate joint basking.

The Valentin’s rock lizard is characterized by unique morphological and ecological adaptations to extreme habitats on high elevations. It was active at low ambient temperatures (Figure 11) and had a relatively large body size in comparison with that of other species of rock lizards. The female-biased sexual size dimorphism has been reported previously (Arakelyan, 2002; Darevsky, 1967) and is supported by our data. Unlike in other small lacertids with a similar lifespan where females grow faster (Kolarov et al., 2010), the growth trajectory in male and female Valentin’s lizard before the fourth winter was similar; however, after this, only females continued to grow (Figure 8; Kurnaz et al., 2017). The maximum body length for females was 79 mm, which is larger than that recorded for *D. valentini* from the highland population in Balahor (Turkey, 2 400 m a.s.l.) (Kurnaz et al., 2017). The body size of female Valentin’s lizard is close to the upper limits reported for rock lizards. Only triploid hybrids between parthenogenetic and biparental species exceeds 80 mm in body length (Arakelyan, 2002).

Valentin’s lizards occupy areas with stable cold climates, where minus temperatures are common from December to March and cold nights are customary from October to April (Figure 2). Larger body size of species living in cold climates has been described for lizards, although it is not a steadfast rule (Pincheira-Donoso et al., 2008) and the weight/SVL proportion does not appear to be affected by mean environmental temperatures (Meiri, 2010). Smaller species of rock lizards (e.g., *D. caucasica*) are also known to exist at such elevations. Such lizards generally shelter within narrow crevices between rocks, deep enough to maintain stable positive temperatures (Darevsky, 1967); however, such shelters are too small for larger individuals, who are thus more likely to die during cold winters. This might be a possible explanation for the arrested growth in the studied species. Arrested growth also affects the results of skeletochronological study and reduces maximal age of the animals due to absence of new marks of growth. That is why previous studies suggest a lifespan of seven years for this species, as estimated by skeletochronology (Arakelyan, 2002). The average lifespan of biparental and parthenogenetic rock lizards has been estimated at five to six years (Arakelyan & Danielyan, 2000). Our observations support this suggestion, although a prolonged lifespan of nine years is possible for *D. valentini* from Turkey (Kurnaz et al., 2017) and parthenogenetic *D. armeniaca* (Galoyan, 2013a). Still, the only way of evaluating the actual lifespan of long-lived reptiles is via prolonged visual observations. According to Tsellarius & Tsellarius (2009), the lifespan of Brauner’s rock lizard is 14 years and might differ between residential and non-residential as well as territorial and wandering animals. Our observations suggest that male Valentin’s lizards have a shorter life span than that of females (Figure 6), although this should be clarified in future studies.

The reproductive period differs among species of rock lizards (Darevsky, 1967). We observed first matings in Valentin’s rock lizard just after emergence from winter hibernation shelters in mid-May. Some other species (*D. brauneri*) start mating after two or three weeks of activity (Tsellarius & Tsellarius, 2001). The gonadal ripening and mating period in *D. raddei nairensis* in Lchashen occur one month later than that in *D. valentini* in the same locality (Danielyan, 1965). Our observations indicate that *D. portschinskii*, which occurs on the steppes at lower elevations of 1 500 m a.s.l. (Arakelyan et al., 2011), started to reproduce in early spring (May) within a short time after emergence, unlike *D. raddei* with which it coexists (own observations). Early reproduction might be an adaptation to harsh conditions at high elevations, but this suggestion should be tested among different species.

According to our findings and those of Darevsky (1967), female Valentin’s lizards have, on average, five to six eggs per clutch (Table 6; Darevsky, 1967), reaching, according to our data, up to 12 eggs. Egg size in Valentin’s lizard was larger than that in most related species at 14–15 mm×8–9 mm (Darevsky, 1967; unpublished data), although smaller than that in *D. zchzerbaki* and *D. rudis macromaculata* (Table 36 in Darevsky, 1967), which are not high-elevation species. Hence, egg size can’t be the obvious adaptation for the living at the high elevation conditions as well.

The high abundance of diverse prey in the meadows (unpublished data) is comparable with that in deciduous forests (Tsellarius & Tsellarius, 2001). The high taxonomic variety of prey items indicated diverse foraging tactics (Figure 9). Valentin’s lizards are active foragers, mostly searching for prey in a cruising foraging mode: animals catch prey in the basking area or on the move. Flies of the Bombyliidae and Sarcophagidae families were common prey items, which were caught in mid-jump from stems or even directly in flight. Hunting juveniles also requires good reactions. The high proportion of immobile objects in their diets, such as larvae, further demonstrated that Valentin’s rock lizards are good searchers and would often find such prey under stones and in the grass. Hence, this species is a universal forager with a great variety of prey items and searching strategies.

Different species or unisexual and biparental rock lizards demonstrate a similar preferred body temperature of about 31–32°С, although they inhabit biotopes with different temperature and light conditions: e.g., dense deciduous forests or highland meadows (Galoyan, 2010; Tsellarius & Tsellarius, 2001; Table 6). Primarily, Valentin’s lizards achieved their relatively high body temperature by basking, a common behavior for poikilothermic reptiles (Pianka & Vitt, 2003) and especially relevant in the cold climate highlands. Such conditions explain their late emergence (1000–1100 h) and mid-day activity peak in spring. The breeding season coincidences with this period, with males then able to find females above the surface for mating and communication. This became almost impossible when temperatures increased in mid-summer (Figures 5, 6). Besides direct basking, Valentin’s rock lizards also use the heat from stones and aggregate together on the stony mounds in joint basking, often with physical contact. Several females of *D. valentini* cooperated in such a joint basking. A single male could join them, however, two and more males were never observed to bask close to each other. Such joint basking might help increase body temperatures in cold climates. Because the relatively small and compact home ranges often overlapped, the basking areas of individuals also often coincided.

Although we found no differences in body temperatures between males and females, the latter were more dependent on substrate temperature (Figure 11). This may be explained by lower activity (also indicated by smaller range sizes) in females. As prey items were abundant around basking sites, the main reason for male activity was mate searching and guarding, and they could achieve high body temperatures by metabolic heat from muscles during movement.
